# The complex interplay between psychological factors and sports performance: A systematic review and meta-analysis

**DOI:** 10.1371/journal.pone.0330862

**Published:** 2025-08-26

**Authors:** Mert Ayranci, Mehmet Kemal Aydin

**Affiliations:** Faculty of Sport Sciences, Hitit University, Corum, Türkiye; Portugal Football School, Portuguese Football Federation, PORTUGAL

## Abstract

The increasing body of research underscores that athletic performance is not solely contingent upon physical capabilities but is also significantly influenced by psychological strengths. Despite this, there remains a need for comprehensive meta-analyses to rigorously investigate the link between psychological factors and sports performance. In line with this, the present study seeks to examine the influence of various psychological constructs, such as motivation, self-efficacy, self-confidence, goal setting, attention, stress management, extraversion, self-discipline, personality traits, and emotional intelligence (EI) on athletic performance. This analysis was conducted in accordance with PRISMA guidelines and included 127 studies covering a total of 24,358 participants. The methodological quality of the included studies was assessed using the Joanna Briggs Institute (JBI) Critical Appraisal Checklist, ensuring a rigorous evaluation of study designs and data reliability. The findings indicate an association between personality traits—such as motivation (d = 0.525), self-efficacy (d = 0.413), conscientiousness (d = 0.316), and extraversion (d = 0.336)—and sports performance. Moreover, the overall association between psychological factors and sports performance was calculated as moderate (d = 0.329). Moderator analyses revealed no significant associations based on variables such as gender, type of sport, or type of athlete. Additionally, no significant associations were found for anxiety, openness to experience, neuroticism, or agreeableness, suggesting that these traits may have more complex or context-dependent relationships with performance. The findings of this meta-analysis indicate that psychological skills training plays a critical role in enhancing athletes’ performance. Future research should delve deeper into studies conducted in specific contexts to better understand the ambivalent relations among these factors.

## Introduction

Over the years, sport psychology has become a major focus of interest in understanding the various psychological factors that influence athletic performance. Among these factors, concepts such as motivation, self-efficacy, self-confidence, goal setting, attention, stress management, anxiety, personality, and emotional intelligence have become particularly salient [1,2]. Although the link between these factors and sport performance has been extensively examined in the literature, there remains a critical need for further research into their practical application and the role they play in mapping the psychological profiles of athletes [3–6]. Motivation and self-efficacy play important roles in optimizing athletes’ performance [7,8]. However, there are still gaps in the measurement and applicability of these psychological factors [9]. In individual sports, self-efficacy and motivation are closely tied to conscientiousness and mental resilience, whereas in team sports, social support and collaboration play a more significant role in enhancing performance [10,11]. This study aims to conduct a meta-analysis focusing on the associations between psychological constructs and sport performance, as these relationships have been widely examined in existing literature [12,13].

In recent years, the important roles that emotional intelligence and personality traits play in sport performance, especially in the management of negative emotional states such as stress and anxiety, have been increasingly recognized [14,15]. Particularly in individual sports like archery or tennis, emotional intelligence has been shown to play a critical role in managing stress, enhancing focus, and maintaining composure under pressure [16]. In this context, developing a psychological map of athletes can help coaches and athletes to better understand and manage these factors [17,18]. This study aims to present a systematic review of the existing literature, providing insights that can guide future research and field practice [8,19].

Psychological factors affecting sport performance have long been a topic of discussion in the sport psychology literature. These factors include motivation, self-efficacy, self-confidence, goal setting, attention, stress management, anxiety, openness to experience, neuroticism, agreeableness, extraversion, conscientiousness, personality and emotional intelligence (EI) [20]. However, accurately identifying the associations between these factors and sport performance, as well as understanding how these associations can be applied in practice, remains a significant challenge [21]. Meta-analytic studies have demonstrated that these factors are critically linked to performance enhancement, yet further methodologically rigorous research is required to explore these associations in greater depth [22,23]. In team sports, psychological factors such as cooperation and social cohesion are essential, while individual sports often require higher levels of self-regulation and stress management [24]. For example, self-efficacy and self-confidence have been found to be directly associated with athletes’ performance; however, the strength of these associations may vary across different types of sports and levels of competition [22]. Furthermore, psychological factors such as emotional intelligence, coping strategies, and mental toughness have also been identified as important contributors to performance [23]. This study aims to better understand the psychological dynamics of sport performance and develop strategies to optimize performance by mapping the psychological profiles of athletes. The hypotheses of the study are formulated as follows:

Hypothesis 1: There is an association between psychological factors including motivation, self-efficacy, self-confidence, goal setting, attention, stress management, anxiety, openness to experience, neuroticism, agreeableness, extraversion, conscientiousness, personality traits, and emotional intelligence (EI) and sport performance.

Hypothesis 2: Gender (male/female), athlete type (Elite/Non-elite), and sport type (Team/Individual) moderate the association between psychological factors and sport performance.

## Materials and methods

This meta-analysis examines the associations between psychological factors and sports performance. It was conducted in accordance with the Preferred Reporting Items for Systematic Reviews and Meta-Analyses (PRISMA) guidelines, which are widely accepted for such studies (S1 File-PRISMA 2020 Checklist). The data files (S2 File-Descriptive Data File) collected for the meta-analysis have been registered on the Zenodo open science platform, supported by international institutions, under the DOI number https://doi.org/10.5281/zenodo.13943969. This meta-analysis, based solely on published studies, received a waiver from the Hitit University Non-Interventional Ethics Committee as it did not require ethical approval. Since the study utilized archival data, no direct interaction with human participants was involved.

The quality and reliability of the studies included in the research were evaluated using the Joanna Briggs Institute (JBI) Critical Appraisal Checklist for observational studies. The JBI checklist was employed to systematically assess key elements of study design, methodology, data collection, and statistical analysis, ensuring a structured evaluation of methodological rigor (S3 File-JBI Table). This approach enabled a thorough assessment of study validity, reliability, and potential sources of bias, thereby enhancing the overall interpretability and robustness of the findings. By utilizing JBI’s evidence-based framework, this study ensures a rigorous and transparent evaluation of the included research, contributing to a more reliable synthesis of the available evidence [25].

### Search protocol

At the outset of the study, MA and MKA independently reviewed relevant databases and conducted preliminary research. Following this initial exploration and database review, key concepts thought to potentially linked to sports performance (e.g., motivation, anxiety) were identified. After reaching a consensus on these concepts, the databases were finalized. The finalized databases were Web of Science (WOS), Scopus, and SPORTDiscuss. The specific details of our search strategies within these databases are as follows:

“Sport* performance” AND “motivation”, “Sport performance” AND “self-efficacy”, “Sport performance” AND “self-regulation”, “Sport performance” AND “self-confidence”, “Sport performance” AND “focus” OR “concentration”, “Sport performance” AND “goal setting”, “Sport performance” AND “anxiety management”, “Sport performance” AND “resilience” OR “mental toughness”, “Athlete performance” AND “motivation”, “Athlete performance” AND “self-efficacy”, “Athlete performance” AND “self-regulation”, “Athlete performance” AND “self-confidence”, “Athlete performance” AND “focus” OR “concentration”, “Stress management” AND “sport performance”, “Psychological factors” AND “sport performance”,”Sport Performance” AND “Emotional Intelligence”, “Sport Performance” AND “Personality”, “Sport Performance” AND “Big Five Personality”, “Performance anxiety in sports”

### Data search exclusion and inclusion criteria

In this study, a comprehensive literature review was conducted to examine the associations between psychological factors and sports performance. The searches were conducted in the Web of Science, Scopus, and SPORTDiscuss databases, covering articles published from January 2014 to August 1, 2024. The primary aim of our study was to identify the psychological factors affecting sports performance and to analyze the interplay between sports performance using quantitative methods.

During the search process, titles, abstracts, and keywords were screened based on the following inclusion criteria: (a) the study employed quantitative methods; (b) it examined the relationship between psychological factors such as motivation, self-efficacy, self-confidence, goal setting, attention, stress management, anxiety, openness to experience, neuroticism, agreeableness, extraversion, conscientiousness, personality, and emotional intelligence (EI) and sports performance; (c) the sample consisted of participants involved in sports competitions; and (d) the study presented an effect size or a correlational analysis.

Studies meeting these criteria were selected in accordance with the PRISMA (Preferred Reporting Items for Systematic Reviews and Meta-Analyses) guidelines [26,27]. The research resulted in a total of 32,541 studies. Additionally, references from previous meta-analyses and reviews on the identified psychological factors and sports performance were examined [28–31]. Publication bias was assessed using visual and statistical methods [32,33].

**Fig 1:**
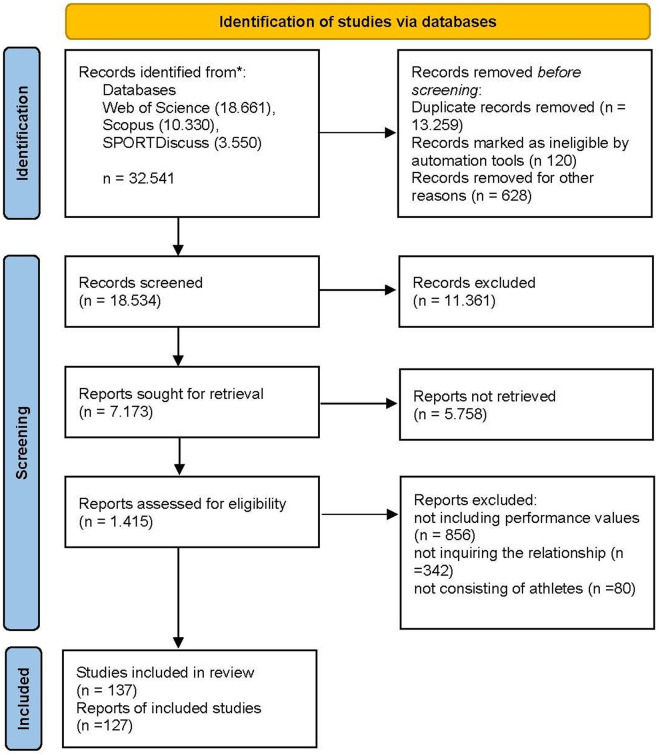
PRISMA flow chart for the identification of the included studies [27].

The selection of studies based on inclusion and exclusion criteria was conducted independently by three authors, with disagreements reviewed and resolved by senior authors. After the abstract review, potentially eligible studies underwent full-text review to determine final eligibility for inclusion. The figure related to the PRISMA guidelines is provided Fig 1.

### Coding of studies and quality

After the data were collected, they were coded to determine their inclusion in the study and to conduct the analyses. For this purpose, we examined the measurement tools from which the identified psychological factors were derived and reported the correlation levels reported within these tools. To avoid publication bias, we also reviewed studies that were not found to be statistically significant. If personality results were reported through sub-dimensions, we presented the associations based on these sub-dimensions; if a total score or overall association was reported, we proceeded with the overall association.

The descriptive findings of the studies were coded into three groups based on gender, type of sport, and athlete level. For example, we categorized them as team sports, individual sports, or both. The criteria for measuring sports performance included self-reports from athletes, self-reports from coaches, and training and competition results. For the examination of publications, the studies were divided into two groups based on publication years to ensure a homogeneous distribution: 2014–2019 and 2020–2024. After gathering the studies, the number of citations on Google Scholar was counted by the researchers. Throughout this process, the findings of the studies were independently coded by two researchers (MKA and MA), and their ratings were compared. In cases of disagreement, a third evaluator (MK) was consulted, and any discrepancies were resolved through discussion and consensus.

To assess the quality of the included studies, the Joanna Briggs Institute (JBI) Critical Appraisal Checklist for observational studies was used. The JBI checklist provides a structured framework for evaluating the methodological quality of studies by assessing aspects such as sample appropriateness, measurement reliability, validity, control of confounding factors, and statistical analysis. The checklist consists of multiple items that address the rigor and transparency of study design and reporting.

Each study was assessed independently by two researchers (MKA and MA), and discrepancies were resolved through discussion. Studies were rated based on their adherence to JBI criteria, with a scoring system ranging from 0 to 8, where higher scores indicate greater methodological rigor. In cases where missing data were identified, the researchers contacted the study authors via email to request additional information. No studies were excluded solely due to missing data.

A total of 137 research articles were evaluated (S2 File-Descriptive Data File). After applying the JBI quality assessment, 10 studies were excluded from the analysis as they did not meet the necessary inclusion criteria. Reasons for exclusion included reporting data solely as descriptive statistics, failure to provide sample size (n), and insufficient methodological detail to allow for meaningful statistical analysis.

In this study, the inter-rater reliability between the two raters was assessed using Cohen’s Kappa statistic. The Kappa value was found to be 0.68, indicating a good level of agreement between the raters. This suggests that the coding and classification process was performed with a high degree of consistency, minimizing the potential for subjective bias. The findings of the studies are presented in the table below.

### Statistical analysis

In this study, after coding all the data and determining that they met the inclusion criteria for analysis, the prepared data were transferred to the JAMOVI 2.5 software package, where final checks were conducted. The appropriate analysis methods for the data were then determined. Using the MAJOR add-on within the Jamovi software package, the correlation coefficient was calculated based on the author names, sample sizes (n values), and r values of the studies. A random effects model was also employed to interpret the findings [34,35]. This model assumes that error arises not only from sampling procedures but also from additional between-study variance [35,36]. In analyses conducted using this method, effect sizes are adjusted by the inverse of the variance’s weight to account for both sampling error and between-study error [37]. The effect sizes within the study were calculated according to Cohen’s guidelines [38]. The I-square (I²) value within the study estimates the degree of overlap in confidence intervals and is interpreted as low (25%), moderate (50%), or high (75%) levels of total variance attributable to covariates [39]. A high I² value indicates significant heterogeneity and justifies the use of a random effects model for meta-analysis [40].

Meta-analyses are frequently vulnerable to publication bias, where studies with significant results are more likely to be published, potentially distorting the overall effect size when combining results from multiple studies [35,36,41]. To mitigate this issue, we examined the symmetry of the effect distribution by visually inspecting funnel plots and conducting Begg and Mazumdar [42] regression tests [43,44]. Additionally, we employed trim-and-fill analyses [45] to estimate the number of potentially missing studies and their potential impact on the overall meta-analytic effect. Each study reported the number of participants, the effect size (Pearson’s r), the confidence interval (lower and upper limits), the relative weight, the residual value, and the summary effect size if the study was excluded from the analysis. Furthermore, Microsoft Excel was used to visualize descriptive statistics. When conducting subgroup analyses based on gender, athlete type, and sport type, appropriate criteria were adopted, and the analyses were included accordingly.

## Results

### Characteristics of the studies

In the meta-analysis conducted, 127 studies reporting effect sizes and relationships obtained with 24,358 participants were included in the analysis. The studies included in the analysis considered variables such as the number of participants, gender, type of athlete, type of sport, and research methods employed. The characteristics of the studies are presented in Table 1.

**Table 1:** Characteristics of Studies.

Authours (Year)	N	r Heat Value	Gender	Athletes Type	Sports Type	Research Design
[46] Lourenço et al. (2022)	447	0,43	Male/Female	Elite/Non Elite	Team/Individual	Survey
[47] Rumpf, et al. (2014).	1075	0,85	Male/Female	Elite	Team	Survey
[48] Olmedilla et al. (2018)	129	0,82	Male/Female	Elite/Non Elite	Individual	Survey
[49] Bochaver et al. (2023)	355	0,34	Male	Elite	Team/Individual	Survey
[50] Guinoubi et al. (2023).	114	0,46	Male/Female	Non Elite	Individual	Survey
[51] Granero-Gallegos et al. (2017)	159	0,91	Male/Female	Elite	Team	Survey
[52] Ruiz-Esteban et al. (2020).	134	0,36	Female	Elite	Team	Survey
[53] Wijayanti et al. (2024).	121	0,44	Male/Female	Elite	Individual	Survey
[10] Wibowo (2024).	250	0,30	Male/Female	Non Elite	Individual	Survey
[54] Knoblochova et al. (2021)	128	0,25	Male/Female	Non Elite	Team	Survey
[55] Brozovich-Neyra et al. (2024).	200	0,89	Male/Female	Elite	Individual	Survey
[56] Kaplanova, A. (2024).	114	0,09	Male	Non Elite	Team	Survey
[57] Neldi et al. (2023).	17	0,93	Male	Non Elite	Individual	Mixed
[58] Quintero-Ovalle et al. (2023).	317	0,14	Male/Female	Elite	Team/Individual	Survey
[59] Kargapolova et al. (2022)	168	0,31	Male/Female	Elite	Individual	Survey
[60] Benítez-Sillero et al. (2021).	118	0,41	Male	Elite	Team	Survey
[61] Almagro et al. (2020).	339	0,35	Male/Female	Elite	Team/Individual	Survey
[62] Javed et al. (2020).	510	0,41	Male/Female	Elite	Individual	Survey
[63] de Oliveira Castro et al. (2020)	92	0,40	Female	Non Elite	Team	Mixed
[64] Espada & Fradejas (2019)	816	0,28	Male/Female	Elite	Team/Individual	Survey
[65] Blecharz et al. (2015)	197	0,18	Male/Female	Non Elite	Individual	Survey
[66] Hartoto et al. (2023)	295	0,14	Male/Female	Elite	Individual	Survey
[67] Ihsan et al. (2022)	30	0,31	Male	Non Elite	Individual	Survey
[54] Knoblochova et al. (2021).	128	0,26	Male/Female	Elite	Team	Survey
[68] Nuț et al. (2017).	58	0,75	Male/Female	Non Elite	Individual	Survey
[69] Koka et al. (2020)	128	0,37	Male/Female	Elite	Individual	Survey
[70] Behzadnia et al. (2018)	140	0,22	Male/Female	Elite	Individual	Survey
[71] Bingöl & Yıldız (2021)	325	0,25	Male/Female	Elite	Individual	Survey
[72] Aizava et al. (2023)	77	0,30	Male	Non Elite	Team	Survey
[73] Baretta et al. (2017)	129	0,27	Male/Female	Elite	Individual	Survey
[74] Raman & Rajaraman (2023)	42	0,41	Male/Female	Elite	Team	Mixed
[75] LaForge-MacKenzie & Sullivan (2014)	47	0,34	Male/Female	Elite/Non Elite	Team/Individual	Mixed
[76] Beattie et al. (2017)	87	0,40	Male/Female	Elite	Individual	Mixed
[77] Lee et al. (2021)	187	0,40	Male/Female	Elite/Non Elite	Team/Individual	Survey
[65] Blecharz et al. (2014)	30	0,35	Male	Non Elite	Team	Mixed
[78] Bosma & Van Yperen (2020)	141	0,15	Male/Female	Elite	Team	Survey
[79] Rouquette et al. (2021)	205	0,36	Male/Female	Elite	Team	Survey
[66] Hartoto et al. (2023)	295	0,25	Male/Female	Elite	Individual	Survey
[80] Pandey & Khusboo (2024)	207	0,36	Male/Female	Elite	Individual	Mixed
[81] Wang et al. (2022)	232	0,13	Male	Elite	Individual	Survey
[82] Teques et al. (2019).	845	0,00	Male/Female	Elite/Non Elite	Team	Survey
[10] Wibowo (2024)	250	0,28	Male/Female	Non Elite	Individual	Survey
[83] van Raalte & Posteher (2019)	459	0,51	Male/Female	Elite	Team/Individual	Survey
[84] Estevan et al. (2014)	50	0,97	Male/Female	Non Elite	Individual	Mixed
[85] Molina et al. (2017)	100	0,73	Male/Female	Elite	Individual	Survey
[86] Zsheliaskova-Koynova (2017).	35	0,49	Male/Female	Elite	Individual	Survey
[87] Castillo-Rodríguez et al. (2023).	27	0,40	Male/Female	Elite	Team	Mixed
[88] Pettersen et al (2023).	156	0,19	Female	Elite/Non Elite	Team	Mixed
[89] Ahmad et al. (2023)	32	0,36	Male/Female	Non Elite	Individual	Mixed
[90] Nassib et al.(2022)	18	−0,76	Male/Female	Non Elite	Individual	Mixed
[91] Znazen et al. (2017)	40	0,77	Male/Female	Non Elite	Individual	Mixed
[92] Fabbricatore et al. (2023)	161	0,60	Male/Female	Elite	Individual	Survey
[93] Brace et al. (2020)	56	−0,25	Male/Female	Non Elite	Individual	Survey
[94] Zarić et al. (2021)	38	0,34	Female	Elite	Team	Survey
[95] Laborde et al. (2014)	28	0,33	Male/Female	Elite	Individual	Mixed
[96] Di Corrado et al. (2014)	47	0,08	Male/Female	Elite	Team/Individual	Survey
[97] Malanov & Subaeva (2021)	41	0,35	Female	Non Elite	Individual	Mixed
[98] Lehner & Schuster (2023)	50	0,41	Male/Female	Elite	Team	Survey
[99] Iwasaki et al. (2017)	281	0,16	Male/Female	Elite/Non Elite	Team/Individual	Survey
[91] Znazen et al. (2016).	40	0,57	Male/Female	Non Elite	Individual	Survey
[92] Fabbricatore et al. (2023)	161	0,75	Male/Female	Elite	Individual	Survey
[96] Di Corrado et al. (2014)	47	0,16	Male/Female	Elite	Team/Individual	Survey
[81] Wang et al. (2022)	61	0,34	Male	Elite	Individual	Survey
[100] Firmansyah et al. (2024)	37	0,35	Female	Elite	Individual	Survey
[101] Wu et al. (2021)	101	0,20	Male/Female	Elite	Individual	Survey
[102] Glavaš, D. (2020).	46	−0,09	Male	Elite	Team	Survey
[103] Coman, P. (2015).	23	0,36	Male	Non Elite	Team	Survey
[104] Mitić et al. (2020)	340	0,10	Male/Female	Elite	Team/Individual	Survey
[105] Pété et al. (2023)	15	0,28	Male	Non Elite	Individual	Survey
[106] Laborde et al. (2014)	973	0,31	Male/Female	Elite	Team/Individual	Survey
[107] Moen et al. (2016)	57	−0,36	Male/Female	Non Elite	Team/Individual	Mixed
[108] Daly et al. (2023)	50	−0,82	Male/Female	Elite/Non Elite	Team	Survey
[109] Lee et al. (2022)	106	−0,37	Male/Female	Non Elite	Individual	Survey
[95] Laborde et al. (2014).	28	0,19	Male/Female	Elite	Individual	Mixed
[89] Ahmad et al. (2023)	32	0,31	Male/Female	Non Elite	Individual	Mixed
[92] Fabbricatore et al. (2023)	161	0,36	Male/Female	Elite	Individual	Survey
[110] Klatt et al. (2021)	82	0,03	Male/Female	Non Elite	Team	Survey
[111] Piepiora (2024)	83	0,03	Male/Female	Elite	Individual	Survey
[112] Khan et al. (2016)	135	0,73	Male/Female	Elite	Team	Survey
[113] Gorelová & Halama (2024)	81	0,11	Female	Non Elite	Team	Survey
[114] Zar et al. (2022)	376	0,16	Male/Female	Elite	Team	Survey
[92] Fabbricatore et al. (2023)	161	0,19	Male/Female	Elite	Individual	Survey
[115] Kalinowski et al. (2020)	122	−0,03	Male	Elite	Team	Survey
[110] Klatt et al.(2021)	82	−0,02	Male/Female	Non Elite	Team	Survey
[18] Kim et al. (2020)	51	−0,34	Male	Non Elite	Team	Survey
[111] Piepiora (2024)	83	−0,65	Male/Female	Elite	Individual	Survey
[112] Khan et al. (2016).	135	0,79	Male/Female	Elite	Team	Survey
[113] Gorelová & Halama (2024)	81	−0,25	Female	Non Elite	Team	Survey
[116] Tomczak et al. (2024).	289	0,33	Male/Female	Elite	Team/Individual	Survey
[92] Fabbricatore et al. (2023)	161	0,18	Male/Female	Elite	Individual	Survey
[18] Kim et al. (2020)	51	0,38	Male	Non Elite	Team	Survey
[110] Klatt et al. (2021)	82	0,08	Male/Female	Non Elite	Team	Survey
[111] Piepiora (2024).	83	0,05	Male/Female	Elite	Individual	Survey
[112] Khan et al. (2016).	135	−0,50	Male/Female	Elite	Team	Survey
[113] Gorelová & Halama (2024).	81	0,05	Female	Non Elite	Team	Survey
[116] Tomczak et al. (2024).	289	0,11	Male/Female	Elite	Team/Individual	Survey
[114] Zar et al. (2022)	161	0,47	Male/Female	Elite	Team	Survey
[92] Fabbricatore et al. (2023)	122	0,18	Male/Female	Elite	Individual	Survey
[115] Kalinowski et al. (2020)	82	0,13	Male	Elite	Team	Survey
[110] Klatt et al. (2021)	83	0,26	Male/Female	Non Elite	Team	Survey
[111] Piepiora (2024)	376	0,17	Male/Female	Elite	Individual	Survey
[112] Khan et al. (2016)	135	0,70	Male/Female	Elite	Team	Survey
[113] Gorelová & Halama (2024)	81	0,28	Female	Non Elite	Team	Survey
[88] Pettersen et al. (2023)	156	0,27	Female	Elite	Team	Survey
[116] Tomczak et al. (2024)	289	0,31	Male/Female	Elite	Team/Individual	Survey
[92] Fabbricatore et al. (2023)	161	0,20	Male/Female	Elite	Individual	Survey
[115] Kalinowski et al. (2020)	122	0,19	Male	Elite	Team	Survey
[110] Klatt et al. (2021)	82	−0,09	Male/Female	Non Elite	Team	Survey
[18] Kim et al. (2020).	51	0,90	Male	Non Elite	Team	Survey
[111] Piepiora (2024)	83	0,22	Male/Female	Elite	Individual	Survey
[112] Khan et al. (2016).	135	0,77	Male/Female	Elite	Team	Survey
[117] Perry et al. (2017).	73	−0,14	Male/Female	Elite	Individual	Survey
[113] Gorelová & Halama (2024)	81	0,06	Female	Non Elite	Team	Survey
[88] Pettersen et al. (2023)	156	0,04	Female	Elite	Team	Survey
[116] Tomczak et al. (2024)	289	0,24	Male/Female	Elite	Team/Individual	Survey
[118] Klein et al. (2017)	1399	0,02	Male/Female	Elite	Individual	Survey
[119] Piepiora & Piepiora (2021)	1260	0,62	Male/Female	Elite	Team/Individual	Survey
[120] Habib et al. (2019)	232	0,47	Male/Female	Elite	Individual	Survey
[18] Kim et al. (2020)	51	0,81	Male	Non Elite	Team	Survey
[121] Çeviker et al. (2020)	250	0,61	Male/Female	Elite	Team	Survey
[95] Laborde et al. (2014)	28	−0,25	Male/Female	Elite	Individual	Survey
[106] Laborde et al. (2014)	973	−0,13	Male/Female	Elite	Team/Individual	Survey
[122] Arribas-Galarraga et al. (2017)	386	0,23	Male/Female	Non Elite	Team	Survey
[123] Gómez-García et al. (2022)	60	0,24	Female	Non Elite	Team	Survey
[104] Mitić et al. (2020)	340	0,21	Male/Female	Elite/Non Elite	Team/Individual	Survey
[124] Nakisa & Ghasemzadeh-Rahbardar (2021)	120	0,82	Male	Elite	Team	Survey
[125] Kopp et al. (2021).	323	0,04	Male/Female	Elite/Non Elite	Team/Individual	Survey
Total Research: 127 Total Sample Size: 24.358						
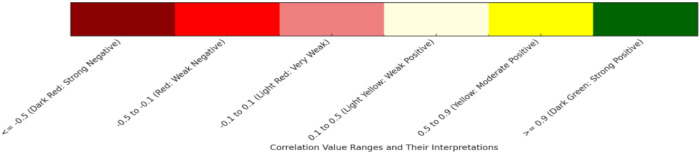						

The characteristics of the studies and their results are presented in Table 2. According to the analysis results, the highest positive correlation (r = 0.97) is attributed to Estevan et al. (2014), while the lowest negative correlation (r = −0.817) is associated with Daly et al. (2023) [83,107]. The median of the observed correlations is (r = 0.25).

**Table 2:** Meta-Analysis Results of Psychological Factors on Sports Performance.

Sport Performance	Sample		Effect Size Statistic				Heterogeneity				Publication Bias
Subgroups	k	N	Estimate (d)	se	p	%95 CI	Tau2	I2	Q	p	Begg and Mazumdar p
Motivation	28	6964	0.525	0.084	<.001	[0.359 – 0.691]	0.191	%97,85	1254.655	<.001	0.027
Self-efficacy	16	3283	0.413	0.079	<.001	[0.256 – 0.569]	0.091	%94,55	275.233	<.001	0.306
Self Confidence	12	738	0.329	0.130	<.012	[0.074 – 0.585]	0.177	%90.7	118.271	<.001	0.545
Goal Setting	6	620	0.460	0.173	<.008	[0.121 – 0.798]	0.161	%93.14	72.937	<.001	0.719
Attention	4	245	0.210	0.096	<0.029	[0.022 – 0.398]	0.018	%49,97	5.996	<.112	1.000
Stress Management	4	1351	0.238	0.082	<.004	[0.077 – 0.400]	0.014	%72,79	12.371	<.006	0.750
Anxiety	5	273	−0.294	0.230	>.202	[-0.745 – 0.158]	0.241	%92,19	50.113	<.001	0.483
Openness to experience	5	542	0.302	0.173	>0.081	[-0.037 – 0.651]	0.139	%93,47	65.489	<.001	0.817
Neuroticism	9	1380	0.042	0.172	>0.804	[-0.294 – 0.380]	0.256	%97,32	225.297	<.001	0.119
Agreeableness	7	882	0.039	0.109	>0.717	[-0.174 – 0.254]	0.072	%89,37	56.460	<.001	0.998
Extraversion	9	1485	0.336	0.073	<.001	[0.192 – 0.480]	0.040	%86,34	58.546	<.001	0.877
Conscientiousness	10	1233	0.316	0.130	<.015	[0.061 – 0.570]	0.158	%94,86	175.129	<.001	0.601
Personality	5	3192	0.607	0.179	<.001	[0.256 – 0.957]	0.154	%98,74	389.372	<.001	0.817
EI	7	2230	0.225	0.136	<.001	[0.045 – 0.128]	0.120	%96,99	199.439	<.001	0.837
TOTAL	127	24.358	0.329	0.036	<.001	[0.258 – 0.400]	0.152	%96,6	3702.968	<.001	0.495

**Fig 2:**
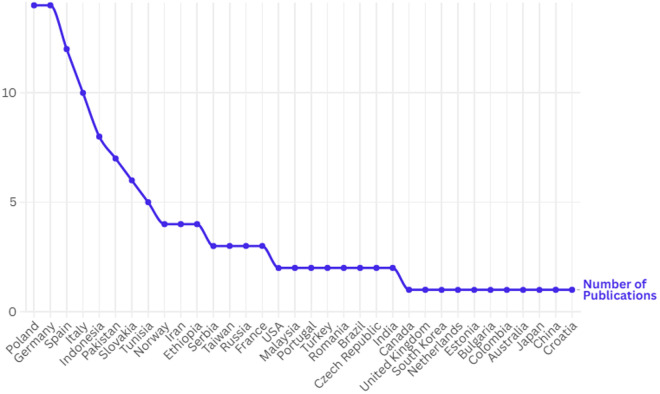
Distribution of publications by country.

Additionally, the countries where the studies were conducted have been provided as supplementary information, and a line chart has been created to illustrate the number of studies conducted in each country. The chart presented in Fig 2 demonstrates the distribution of publications across various countries, highlighting those with the highest number of studies.

According to the analysis results, the leading with the most publications is Germany (14) and Poland (14), followed by the following countries: Spain (12), Italia (10), Indonesia (8), Pakistan (7), Slovakia (6), Tunisia (5), Ethiopia (4), Norway (4), Iran (4), France (3), USA (2), Russia (3), Serbia (3), Taiwan (3), Brazil (2), Czech Republic (2), India (2), Malaysia (2), Pakistan (2), Portugal (2), Romania (2), Turkey (2), Australia (1), Bulgaria (1), Canada (1), China (1), Colombia (1), Croatia (1), Estonia (1), Japan (1), Netherlands (1), South Korea (1), United Kingdom (1).

Table 2 provides a detailed overview of the effect sizes of psychological factors on sports performance (H1) as reported in various studies. It presents a comparative analysis of how each psychological factor is associated with athletes’ performance, highlighting the strength of these associations. The data summarized in the table offer insights into the nature whether positive or negative of these associations with performance, drawing on findings from research in the field of sports psychology. Furthermore, Table 2 presents the heterogeneity test results and publication bias results related to the associations between psychological factors and sports performance as derived from the meta-analysis.

**Fig 3:**
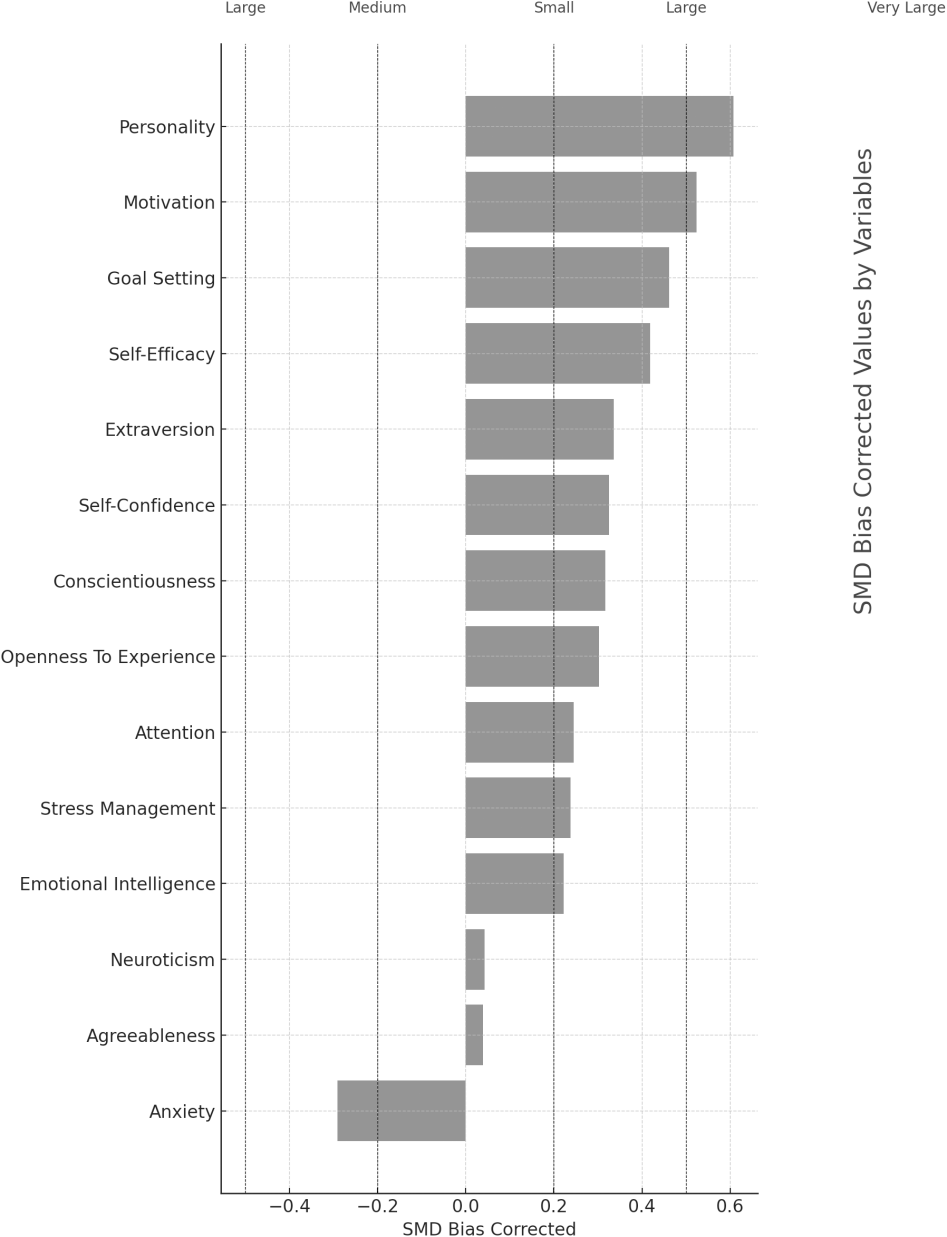
Standardized mean difference (SMD) values by significant categories.

According to the analysis results, the effect sizes on sports performance were as follows: motivation, d = 0.525, 95% CI [0.359–0.691], indicating a moderate positive association; self-efficacy, d = 0.413, 95% CI [0.359–0.691], indicating a moderate positive association; self-confidence, d = 0.329, 95% CI [0.074–0.585], indicating a moderate positive association; goal setting, d = 0.460, 95% CI [0.121–0.798], indicating a moderate positive association; attention, d = 0.210, 95% CI [0.022–0.398], indicating a small positive association; stress management, d = 0.238, 95% CI [0.077–0.400], indicating a small positive association; extraversion, d = 0.336, 95% CI [0.192–0.480], indicating a moderate positive association; conscientiousness, d = 0.316, 95% CI [0.061–0.570], indicating a moderate positive association; personality, d = 0.607, 95% CI [0.256–0.957]; and Emotional Intelligence (EI), d = 0.225, 95% CI [0.045–0.128], indicating a small positive association. Overall, all psychological factors combined showed a moderate positive association on sports performance, d = 0.329, 95% CI [0.258–0.400]. The study results were additionally modeled, and information related to the model is provided in Fig 3. No significant association was observed between other subgroups. According to Begg and Mazumdar’s test, the analysis showed that p > 0.495, indicating no publication bias.

**Fig 4:**
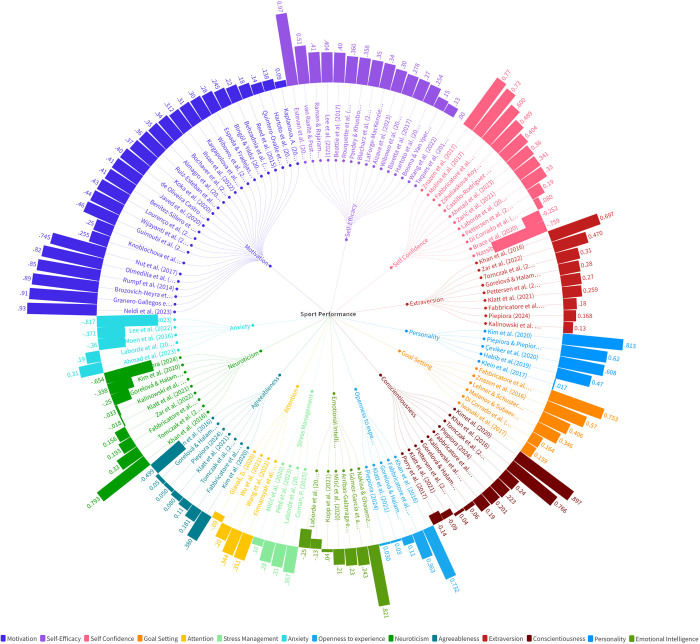
The r values for each study and 14 concepts.

The study results were further modeled to obtain a more comprehensive analysis, and detailed information regarding this modeling is provided in Fig 4. Fig 4 illustrates how the model was structured, which variables were included, and how these variables relate to one another. This modeling allows for a deeper understanding of the study’s findings and facilitates a thorough interpretation of the data obtained. Additionally, the insights provided by the model offer significant support for the overall conclusions of the study.

To summarize and best visualize the resulting effect sizes, correlations were transformed into SMD values (i.e., Cohen’s d). As shown in Table 3 and depicted in Fig 3, Fig 4 we identified 14 concepts that associated withsports performance. The r values for each study and the 14 concepts are illustrated in Fig 4. When assessing the significance of effect size values, 8 association were categorized as small (±0.20 to <0.50) and 2 association as moderate (±0.50 to < 0.80).

**Table 3:** Subgroup analysis results on the association between psychological factors and sports performance.

Sport Performance	Sample		Effect Size Statistic				Heterogeneity	
Subgroups	k	N	Estimate (d)	se	p	%95 CI	I2	Q
Intercept	127	24.358	0.393	0.132	0.003	[0.134 – 0.653]	%96,62	3699.237
Gender			−0.024	0.049	0.614	[-0.121 – 0.072]		
Intercept	127	24.358	0.425	0.089	0.001	[0.250 – 0.600]	%96,52	3595.367
Athletes Type			−0.065	0.055	0.239	[-0.175 – 0.044]		
Intercept	127	24.358	0.388	0.094	0.001	[0.202 – 0.574]	%96,55	3627.175
Sports Type			−0.033	0.049	0.499	[-0.130 – 0.063]		

For values with significant heterogeneity (I² > 25%), we conducted subgroup analyses to examine the association of various psychological factors on sports performance by categorizing studies based on gender, athlete type, and sport type. The analysis revealed that these subgroup comparisons did not show significant differences in sports performance, likely due to the disproportionate number of studies reporting psychological factors.

A forest plot presenting the combined effect sizes from the studies and a funnel plot illustrating the data distribution and potential publication bias are provided (S4 File-Forest and Funnel Plot).

Table 3 presents the results of a meta-analysis examining how subgroup differences are associated with variations in the relationship between psychological factors and sports performance. This analysis explores how various subgroup classifications—such as gender, athlete type, and sport type—moderate the strength and direction of these associations with athletic outcomes, providing a deeper understanding of the conditions under which these associations differ.

## Discussion

In a meta-analysis conducted to gain preliminary insights into the future directions of sport psychology, 127 studies were reviewed over the last decade examining the association of psychological factors such as motivation, self-efficacy, self-confidence, goal setting, attention, stress management, anxiety, openness to experience, neuroticism, agreeableness, extraversion, self-discipline, personality and emotional intelligence (EI) on sport performance. The aim of the study was to determine the extent to which these psychological factors affect athletes’ performance and to provide a guide for coaches and practitioners. The analysis revealed that these factors have a moderate positive association on athletes’ performance (r = 0.329).

The results of this meta-analysis support Hypothesis 1, which proposed that psychological factors such as motivation, self-efficacy, self-confidence, and emotional intelligence would positively influence sport performance; they confirm the critical role of these factors and contribute to the recent literature. Each psychological factor analyzed ‘motivation, self-efficacy, self-confidence, goal setting, attention, stress management, extraversion, conscientiousness, personality and emotional intelligence (EI)’ showed a positive association on sport performance to different degrees. However, the fact that factors such as anxiety, openness to experience, neuroticism and agreeableness did not have a significant association on sport performance is noteworthy and requires further discussion. Motivation was identified as one of the most influential psychological factors and had a moderate effect size (d = 0.525). This finding is in line with the results of recent studies such as Lochbaum et al. (2016) and Clancy et al. (2016) who reported that motivation is a central determinant of sport performance in different contexts [126,127]. Motivation is a key factor not only in sustaining effort and overcoming challenges, but also in promoting goal-setting behaviors, which was found to have a moderate positive association in this analysis (d = 0.460). These findings are consistent with the Deci & Ryan (2000) theoretical framework of self-determination theory and emphasize the importance of motivational strategies in enhancing athletic performance [128–130].

Self-efficacy and self-confidence showed moderate positive association on performance (d = 0.413 and d = 0.329, respectively), which supports the extensive literature emphasizing the importance of athletes’ belief in their own abilities [131,132]. Studies by Chang et al. (2017) and Ahmad et al. (2018) reinforce that self-efficacy is a consistent predictor of athletic success [133,134]. However, the fact that self-confidence has a slightly lower effect size than self-efficacy may be explained by the situational and contextual variability of self-confidence, as discussed by Hays et al. Self-confidence may fluctuate depending on recent experiences and external feedback, which may influence its association on performance [135].

Goal setting emerged with a moderate effect size (d = 0.460), reaffirming the importance of setting specific, challenging goals in improving sport performance [136,137]. The positive association of goal setting has also become the focus of recent research, such as Smith et al. (2019) and Nordin-Bates (2019), which emphasize the importance of structured and systematic goal setting practices in individual and team sports [138,139].

Attention and stress management showed smaller effect sizes (d = 0.210 and d = 0.238, respectively), suggesting their positive but less prominent role in sport performance. These findings are consistent with the complex and context-specific nature of these factors, which has also been noted in studies by Janelle [140] and more recently by Olmedilla et al. [141] Attentional control is critical, especially in high-pressure situations, but its role may vary depending on the specific demands of the sport [142]. Similarly, stress management is associated with sustaining performance under pressure; however, this association may vary depending on individual differences and situational factors [143,144].

Extraversion and conscientiousness also showed moderate positive association (= 0.336 and d = 0.316, respectively), which align with recent findings by Allen et al. (2013) and Smith et al. (2020) [145,146]. The relationship between extraversion and energy, sociability, and positive emotions contributes to its beneficial effect, particularly in team sports and high-risk environments [147]. Conscientiousness is characterized by organization and perseverance, which support consistent training and goal achievement, making it critical for long-term success [148]. These results suggest that personality traits should be considered in athlete development programs for their role in promoting sustainable high performance.

The strongest effect size was found for overall personality (d = 0.607), which strongly supports Hypothesis 2, suggesting that personality traits play a significant role in an athlete’s performance. This result aligns with the broader literature, including studies by Laborde et al. (2016) and more recently Nicholson et al. (2019), which demonstrate that specific personality traits, particularly conscientiousness and emotional stability, are key predictors of performance across various sports [149,150]. These findings emphasize the importance of incorporating personality assessments into athlete selection and development processes to optimize performance outcomes. Despite Emotional Intelligence (EI) showing the smallest effect size (d = 0.225), it still demonstrates a positive role on performance. This result aligns with findings from Zizzi et al. (2003), Cowden (2016), and more recent meta-analyses by Lu et al. (2020), which have shown that EI contributes to effective emotion regulation, team cohesion, and stress management in competitive sports [41,151,152]. The relatively smaller effect size may reflect the complex nature of EI and its context-dependent role, particularly within individual and team sports settings [153].

On the other hand, the findings that anxiety, openness to experience, neuroticism, and agreeableness do not have a significant relationship between sports performance are particularly intriguing and warrant further investigation. The lack of a significant association for anxiety (d = −0.034) is somewhat surprising, given the extensive research highlighting its complex relationship with performance [154,155]. Previous studies have shown that anxiety can be associated with both facilitative and inhibitive outcomes, depending on factors such as the nature of the sport and the athlete’s cognitive appraisals [156,157]. The absence of a significant finding in this meta-analysis may reflect the variability in how different athletes experience and manage anxiety, which could diminish its overall association with sport performance.

Openness to experience, often regarded as a positive trait associated with creative and adaptive thinking [147], also did not show a significant relationship between sports performance in this analysis (d = 0.085). This may be because openness to experience is more relevant in contexts requiring innovation and flexibility rather than in sports, where routine and discipline are typically critical for success. Similar results have been observed in studies by Piedmont et al. (1999) and more recently by Allen et al. (2011), which suggest that while openness to experience contributes to overall personality development, its direct association with athletic performance may be limited [158,159].

Neuroticism (d = −0.025) also did not show a significant association, consistent with mixed findings from previous research on the role of neuroticism across different sports [147,160]. Neuroticism is generally associated with negative emotions and stress, which can negatively link to performance; however, this association may be mitigated by factors such as coping strategies and support systems [161]. The non-significant result in this meta-analysis may suggest that, although neuroticism is often associated with negative outcomes, this association may be mitigated through effective psychological interventions.

Similarly, agreeableness (d = 0.103) did not have a significant relationship between sports performance. This result is consistent with earlier research suggesting that while agreeableness may facilitate teamwork and interpersonal relationships, it may not directly influence performance outcomes in individual or competitive sports [158,159]. The non-significant finding may indicate that agreeableness is more relevant in the context of team dynamics and cohesion, but its association with individual athletic performance may be weaker or less direct.

The combined effect size of all psychological factors showed a moderate positive relationship between sports performance (d = 0.329), which confirms the importance of psychological skills in athletic success. This cumulative effect size underscores the necessity of integrating psychological training into sports coaching and development, reflecting the recommendations of recent comprehensive reviews [162,163].

Furthermore, although the subgroup analyses for gender, athlete level, and sport type did not yield statistically significant effects, this may be attributed to uneven data distribution across categories and the use of generalized psychological measures. These results should be interpreted with caution and considered as methodological limitations rather than definitive evidence of no moderation. The publication bias analysis of the 127 articles included in the study indicated that there was no significant publication bias (p > 0.495), suggesting that the results are robust and reliable. The absence of publication bias supports the validity of the findings and reinforces the generalizability of the positive association of psychological factors on sports performance across different studies.

## Conclusion

In conclusion, this meta-analysis reinforces the critical importance of psychological factors in sports performance. Motivation, self-efficacy, and personality traits have emerged as particularly influential factors, highlighting the necessity of integrating psychological training into sports development programs. However, the non-significant findings for factors such as anxiety, openness to experience, neuroticism, and agreeableness suggest that these variables may exhibit more refined or context-specific associations with sport performance, warranting further investigation.

The findings underscore the importance of a holistic approach to athlete development that includes psychological skills training alongside physical and technical preparation. The breadth of the included studies supports the generalizability of these results, while future research should focus on examining these psychological factors within more specific contexts, such as sports, levels of competition, and cultural environments, to better understand the complex interplay between sports performance.

The insights gained from this analysis provide a foundation for both researchers and practitioners to develop more targeted psychological approaches that take into account the associations between psychological factors and athletic performance. Continued exploration of the complex interactions between psychological factors in sports psychology will further deepen our understanding and support the development of evidence-based practices.

## Practical applications

Despite the moderate effect sizes of psychological variables, particularly factors such as motivation and self-efficacy, their practical applications should not be overlooked. Coaches can enhance motivation by using goal-setting techniques that are tailored to the athlete’s current capacities. Specifically, short-term and attainable goals can help athletes make progress and increase their confidence in their abilities [46]. Self-efficacy, on the other hand, can be reinforced through positive feedback that strengthens athletes’ belief in themselves. In this regard, it is important for coaches to provide constructive feedback and create opportunities for athletes to take small steps toward success [164].

Additionally, when considering the link between emotional intelligence and performance, coaches can teach athletes stress management and mindfulness techniques to develop these skills. Particularly in individual sports, stress management abilities can improve athletes’ performance under pressure [165]. In team sports, social support and team cohesion play a critical role in enhancing both individual and team performance. Coaches who foster collaboration and solidarity within the team can strengthen athletes’ motivation and commitment [51].

These recommendations, as discussed in the previous section, provide practical strategies for coaches and athletes. However, it is important to note that these strategies should be adapted to the individual needs of athletes, and further research is needed to fully explore the interplay between these variables.

## Generalizability of the findings

The findings of this meta-analysis provide valuable insights into the role of psychological factors in sports performance. However, the generalizability of these results should be carefully considered due to several factors. Firstly, while the studies included in this meta-analysis contribute to the robustness of the findings, they also represent a wide range of sports, levels of competition, and participant demographics, which introduces variability. Although the positive association of factors such as motivation, self-efficacy, and personality traits are well-supported, their specific influence may vary across different sports, age groups, and levels of competition.

Additionally, the non-significant association observed for anxiety, openness to experience, neuroticism, and agreeableness suggests that these factors may have context-dependent roles that are not easily captured within a broad meta-analytic framework. For example, the role of anxiety in performance may vary significantly depending on the demands of the sport and the individual athlete’s coping mechanisms. Similarly, personality traits such as openness to experience and agreeableness may be more relevant in the context of specific team dynamics or creative sports, which were not specifically isolated in this analysis.

Furthermore, cultural differences were not extensively examined in this meta-analysis, which could affect the generalizability of the findings. Psychological factors may manifest differently across cultures and potentially influence their impact on performance. Future research should aim to investigate these factors within more specific cultural and contextual frameworks to enhance the applicability of the findings. The absence of publication bias in the study supports the validity of the findings and reinforces the generalizability of the positive association of psychological factors on sports performance across different studies.

## Limitations

While this meta-analysis provides comprehensive insights into the role of psychological factors in sports performance, several limitations should be acknowledged. Firstly, the studies included in the analysis cover a wide range of sports, levels of competition, and participant demographics; this increases the generalizability of the findings but also introduces significant variability. This heterogeneity may obscure the specific associations of psychological factors within sports or competitive contexts [163].

Secondly, the non-significant findings for anxiety, openness to experience, neuroticism, and agreeableness suggest that these factors may exhibit context-specific associations that are not adequately captured within a broad meta-analytic framework. For example, the role of anxiety in sports performance can be both facilitating and inhibiting, depending on the athlete’s cognitive appraisals and coping strategies [154–156]. However, these nuances may be lost when aggregating findings from different studies.

Additionally, cultural differences were not extensively examined, which could limit the applicability of the findings across different cultural contexts. Psychological constructs can vary significantly across cultures, potentially influencing their impact on sports performance [164]. Thus, future research should aim to address these cultural dimensions to enhance the applicability of psychological interventions in sports.

Furthermore, as a limitation, it should be noted that the specific psychological constructs analyzed in this meta-analysis were measured using a variety of tests across different studies. While we focused on studies with robust methodologies, we did not systematically analyze the specific scales used to assess constructs like motivation, self-efficacy, and emotional intelligence. This lack of standardization in measurement tools may have introduced inconsistencies and should be considered when interpreting the results. Future research should include a detailed review of the tests used to measure these constructs to ensure consistency and clarity in the findings. By acknowledging these limitations, we hope to provide a clearer understanding of the scope and boundaries of our meta-analysis, while suggesting areas for improvement in future research.

## Supporting information

S1 File PRISMA 2020 Checklist. (DOCX)

S2 File Descriptive Data File. (XLSX)

S3 File JBI Table. (XLSX)

S4 File Forest and Funnel Plot. (PDF)
