# Supplementary figures and images for: The complex interplay between psychological factors and sports performance: A systematic review and meta-analysis
Source: PLoS One. 2025 Aug 26;20(8):e0330862. doi: 10.1371/journal.pone.0330862 (PMC12380335; doi:10.1371/journal.pone.0330862)

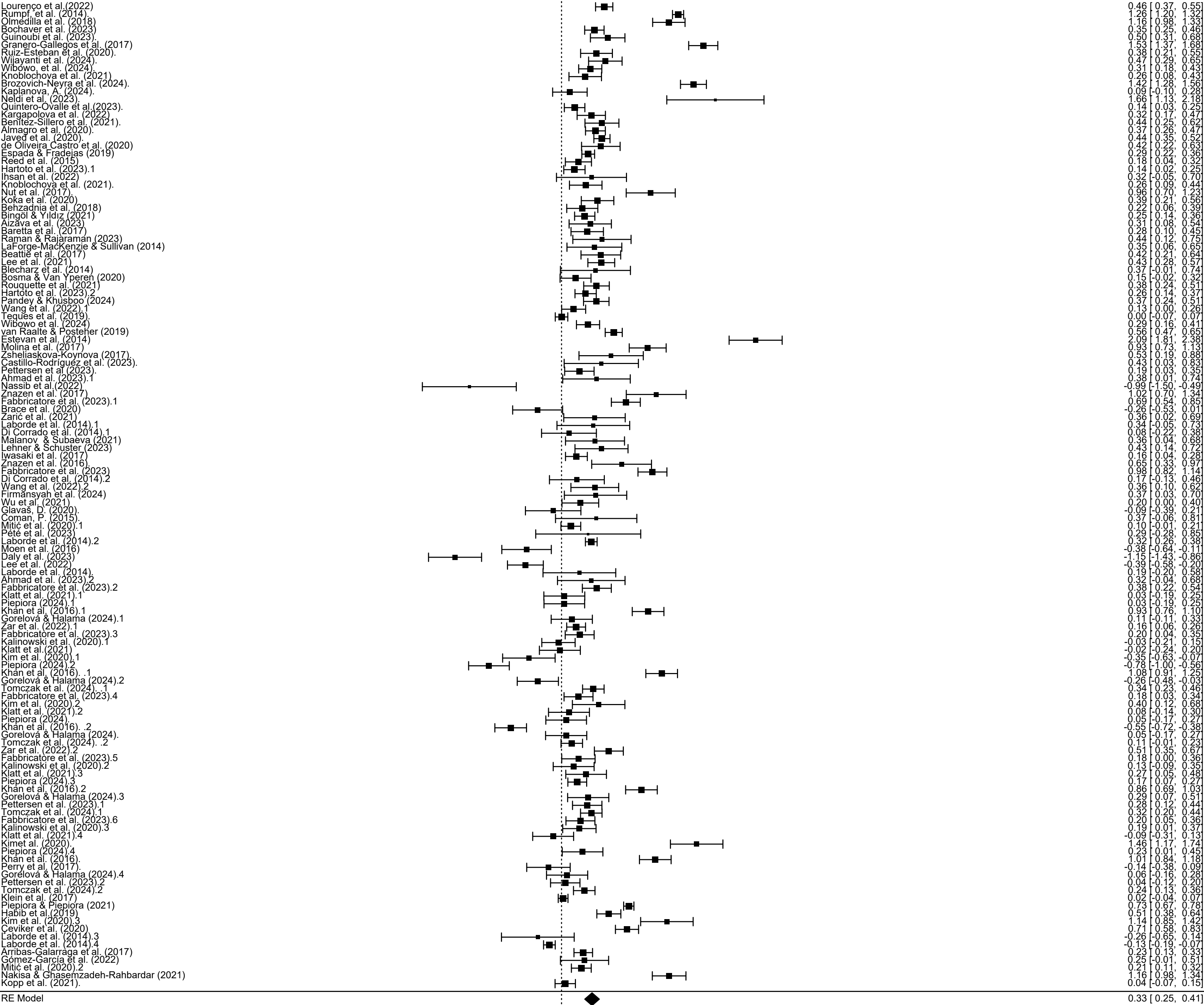

RE Model 0.33 [ 0.25, 0.41]

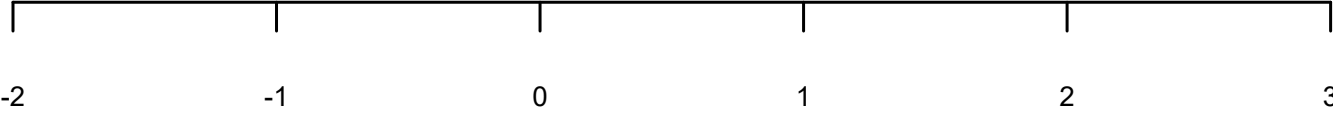

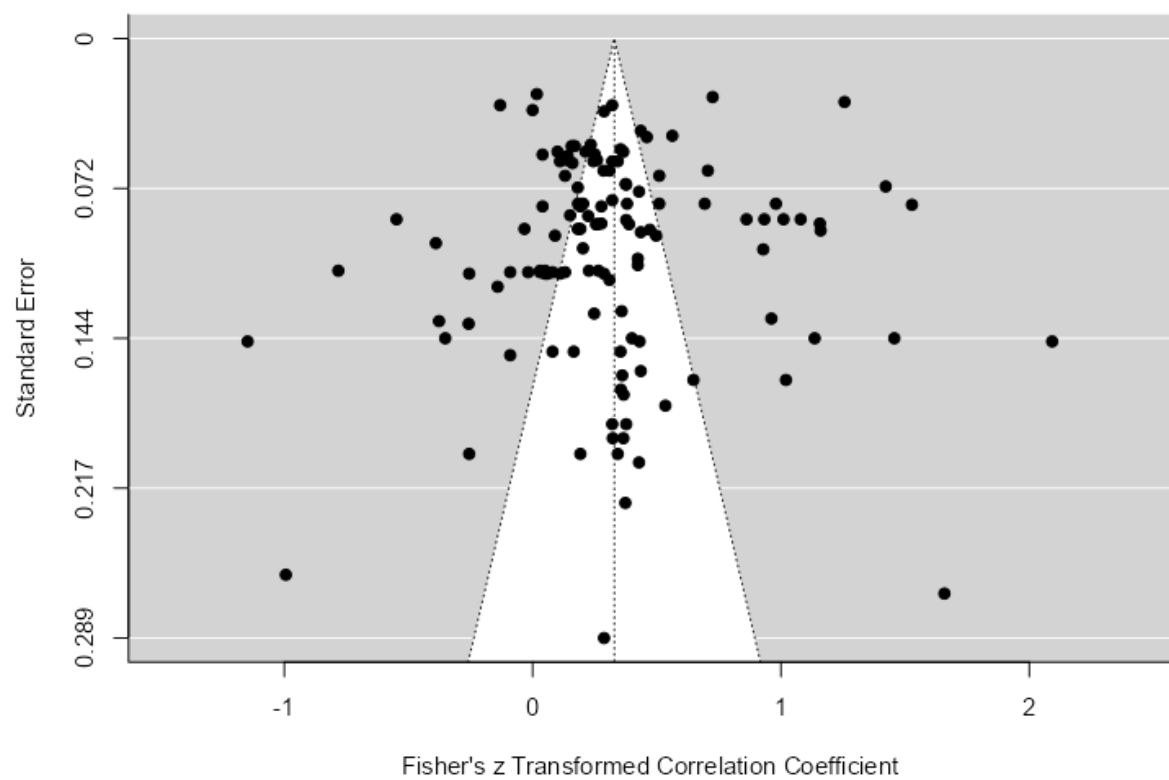

Supplement: S4 File — (PDF) [file pone.0330862.s004.pdf]
